# Diosmetin Induces Modulation of Igf-1 and Il-6 Levels to Alter Rictor-Akt-PKCα Cascade in Inhibition of Prostate Cancer

**DOI:** 10.3390/jcm10204741

**Published:** 2021-10-15

**Authors:** Rebecca Pakradooni, Nishka Shukla, Kalpana Gupta, Jatinder Kumar, Ilaha Isali, Ahmed O. Khalifa, Sanjeev Shukla

**Affiliations:** 1 Department of Urology, Case Western Reserve University, Cleveland, OH 44106, USA; rkp38@case.edu (R.P.); ilaha.isali@case.edu (I.I.); Ahmed.Khalifa@esneft.nhs.uk (A.O.K.); 2 Department of Pathology, Case Western Reserve University, Cleveland, OH 44106, USA; nishkashukla01@gmail.com (N.S.); Kalpana.gupta@case.edu (K.G.); 3 Department of Urology, University of Florida Health Jacksonville, Jacksonville, FL 32209, USA; Kumarj@acmh.org; 4 Department of Urology, ACMH Hospital, 1 Nolte Drive, Kittanning, PE 16201, USA

**Keywords:** prostate cancer, Rictor, AKT, PKCα, diosmetin

## Abstract

Growth signals, which typically originate from the surrounding microenvironment, are important for cells. However, when stimulation by growth factors becomes excessive and exceeds their threshold limit, deleterious effects may ensue. In patients with cancer, maintenance of tumors depends, at least in part, on growth factor stimulation, which can also facilitate cancer progression into advanced stages. This is particularly important when the tumor grows beyond its tissue boundaries or when it invades and colonizes other tissues. These aforementioned malignant events are known to be partly supported by elevated cytokine levels. Among the currently known growth signals, insulin-like growth factor (IGF)-1 and IL-6 have been previously studied for their roles in prostate cancer. Both IGF-1 and IL-6 have been reported to activate the RAPTOR independent companion of MTOR complex 2 (Rictor)/AKT/protein kinase C α (PKCα) signaling pathway as one of their downstream mechanisms. At present, research efforts are mainly focused on the exploration of agents that alter growth factor (such as IGF-1) and cytokine (such as IL-6) signaling for their potential application as therapeutic agents, as both of these have been reported to modulate disease outcome. In the present study, IGF-1 and IL-6 served distinct roles in the androgen responsive LNCaP cell line and in the androgen refractory PC-3 cell line in a dose- and time-dependent manner. Increased phosphorylation of Rictor at the Thr-1135 residue, AKT at the Ser-473 residue and PKCα at the Ser-657 residue were observed after treatment with IGF-1 and IL-6. Subsequently, it was found that diosmetin, a natural plant aglycone, had the potential to modulate the downstream signaling cascade of Rictor/AKT/PKCα to inhibit the progression of prostate cancer. Treatment of LNCaP and PC-3 cells with diosmetin inhibited the phosphorylation of Rictor (Thr-1135), AKT (Ser-473) and PKCα (Ser-657) in a dose-dependent manner. Furthermore, the Bax/Bcl-2 expression ratio was increased in response to diosmetin treatment, which would result in increased apoptosis. Based on these observations, diosmetin may represent a novel therapeutic target for prostate cancer.

## 1. Introduction

The mTOR signaling pathway regulates cell proliferation, survival and metabolism through two distinct protein complexes: mTORC1 and mTORC2 [1]. mTOR has been reported to serve important roles in cardiovascular diseases, aging, diabetes and cancer [2,3,4]. mTOR inhibition by pharmacological or genetic manipulation can extend the lifespan of various animal models. Additionally mTORC1 serves to sense growth signals and nutrients, and dietary restriction results in reduced mTORC1 activity, which in turn promotes longevity [5,6,7,8,9]. Rapamycin, a mTORC1 inhibitor, has been previously revealed to extend the lifespan of yeast, worms, flies and mice [8]. Recent reports suggest that prolonged rapamycin treatment in animals can reduce mTORC2 activity, suggesting the involvement of mTORC2 in lifespan regulation [9,10]. The mTORC2 complex is poorly understood in terms of its role downstream of growth factor signaling activation. The mTORC2 complex consists of four major components, namely mTOR, stress-activated map kinase-interacting protein 1 (SIN1), G protein β subunit-like (GβL) and RPTOR independent companion of MTOR complex 2 (Rictor) [10]. mTORC2 serves a key role in the PI3K/AKT signaling pathway by phosphorylating AKT at Ser-473, which results in its activation. The main component of the mTORC2 pathway is Rictor, which is required for mTORC2 activation. This is supported by previous observations that silencing Rictor expression could inhibit the activation of AKT [11]. In a number of cancer types, the Rictor gene has been found to be upregulated. This includes 18% of neuroendocrine prostate cancers, which is a common type of cancer, 16% of squamous cell lung carcinomas, 12% of sarcomas and 10% of esophagus and stomach cancer cases [12]. Silencing Rictor expression in bone marrow-derived macrophages inhibited cell migration [13], whereas Rictor inhibition has been shown to abrogate IL-4-stimulation, stimulate macrophage M2 polarization and TGF-β1 [14]. Mechanistically, knocking down the expression of mTORC2 components Rictor, Sin1 or mTOR could inhibit the phosphorylation of residues on the turn motifs of both protein kinase Cα (PKCα) and AKT and residues on the hydrophobic motif of AKT. Furthermore, inhibiting components of the mTORC2 complex can decrease the phosphorylation of residues on the hydrophobic motif of PKCα [15]. PKC is a serine/threonine protein kinase that has been demonstrated to regulate a number of cellular processes, including cell migration, cell proliferation and tumor growth [15]. Previous studies have begun to unravel the potential role of mTORC2 in AKT and PKC signaling. mTORC2 can activate PKC, serum/glucocorticoid-regulated kinase (SGK) and AKT by phosphorylation, which in turn mediates cell survival and proliferation [4].

Epidemiological studies have suggested that elevated circulating serum insulin-like growth factor-1 (IGF-1) levels are associated with the development of advanced prostate cancer [16,17]. Overexpression of IGF-1 in the prostate basal epithelial layer of mice resulted in the development of prostate adenocarcinoma, in a similar manner to that seen in human disease [18]. IL-6 has also been shown to be expressed in prostate tumors and in the stromal tumor microenvironment, where it can regulate angiogenesis, apoptosis, proliferation and differentiation [19]. In the prostate, IL-6 paracrine mainly activates two events to promote tumorigenesis, specifically the autocrine IL-6 loop and the autocrine activation of the IGF-1 receptor (IGF-IR) [20].

Since Rictor can activate AKT and PKCα signaling, dysregulation of Rictor can lead to detrimental effects on tumor development. Accumulative evidence suggests that rapamycin can exert potent immunosuppressive properties. By contrast, sirolimus, another inhibitor of mTORC, has been found to cause side effects, including peripheral diarrhea, constipation, edema, hypertension, thrombocytopenia, abdominal pain, headache, hypercholesterolemia, hypertriglyceridemia, fever, nausea, arthralgia, urinary tract infection, anemia, pain and increased creatinine levels [21]. Therefore, we need to have an effective agent that can inhibit cancer progression with less to no toxicity. Diosmetin (5,7,3,’trihydroxy-4’-methoxyflavone) is a natural plant flavone that can modulate the Rictor, AKT and PKCα signaling pathways. It was observed that silencing Rictor expression decreased AKT (Ser-473) phosphorylation, but did not significantly change the phosphorylation of PKCα (Ser-657). Diosmetin treatment of LNCaP and PC-3 prostate cancer cells could also reduce the phosphorylation of AKT (Ser-473) and PKCα (Ser-657) to reduce cell viability. Diosmetin has previously been reported to exert antioxidant, anti-inflammatory and antitumor effects [22], such that it could induce apoptosis in acute myeloid leukemia, HepG2, breast and lung cancer cells. In addition, diosmetin has also been reported to induce apoptosis in prostate cancer cells [23]. Therefore, in the present study, the objective was to assess the effects of diosmetin treatment on the Rictor-associated pathway in prostate cancer, which is the second most common cancer among men.

## 2. Materials and Methods

### 2.1. Cell Lines and Treatments

The androgen-sensitive human prostate cancer cell line LNCaP (cat. no. CRL-1740), androgen-refractory human prostate cancer cell lines PC-3 (cat. no. CRL-1435) and DU145 (cat. no. HTB-81), and the normal prostate epithelial cell line RWPE-1 (cat. no. CRL-11609) were obtained from the American Type Culture Collection, Manassas, VA, USA.

DU145 and PC-3 cells were cultured in RPMI-1640 medium (cat.no. 21875034; Gibco, Waltham, MA, USA) supplemented with 5% FBS and 1% penicillin-streptomycin. RWPE-1 cells were cultured in Keratinocyte Serum-Free Medium Kit (cat. no. 17005-042; Invitrogen; Thermo Fisher Scientific, Inc., Waltham, MA, USA). The kit was combined with bovine pituitary extract and human recombinant epidermal growth factor for proliferation. Monolayer cultures of RWPE-1, PC-3 and DU145 cells were maintained at 37 °C and 5% CO_2_ in a humidified environment. In the cell culture, charcoal-stripped FBS was used only when the cells were treated with IGF-1 (cat.no. 200-06; PeproTech Inc., East Windsor, NJ, USA) at doses of 10, 20, 50, and 100 ng or IL-6 (cat.no. 100-11; PeproTech Inc., NJ, USA) at doses of 1, 5, 10, and 20 ng. At 60% confluence, LNCaP and PC-3 cells were treated with different concentrations of diosmetin (cat. no. D7321; Sigma-Aldrich, St. Louis, MO, USA), dissolved in DMSO, while the control group received equivalent concentrations of DMSO.

### 2.2. Transient Transfection

DU145 and PC-3 cells were seeded into 100-mm culture plates and allowed to attach overnight. The next day, when the cell confluency reached ~80%, DU145 cells were transiently transfected using Lipofectamine 2000 (cat,no. 11668030; Invitrogen; Thermo Fisher Scientific, Inc., Grand Island, NY, USA) with 8 μg pLNCX vector encoding AKT (kindly provided by Professor William Sellers, Broad Institute, Cambridge, MA, USA) or an empty vector.

PC-3 cells were transiently transfected with 8 μg pUSEamp expression vector encoding the dominant negative mutant AKT1 (DN-AKT; Upstate Biotechnology, Inc., Lake Placid, NY, USA). Additionally, PC-3 cells were transfected with Rictor-1410 small interfering RNA (siRNA) encoded within the piLenti-siRNA-GFP vector (cat. no. iV000079a; Applied Biological Materials Inc., Richmond, BC, Canada) using Lipofectamine 2000 transfection reagent (Invitrogen; Thermo Fisher Scientific, Inc., Grand Island, NY, USA). After 6 h of transfection, the medium was replenished with fresh complete medium before the cells were incubated overnight at 37 °C in a humidified incubator. After 48 h of transfection, AKT-overexpressing DU145, DN-AKT-expressing PC-3 and Rictor-knockdown PC-3 cells were washed with PBS prior to the collection of total cell lysates. The cells were then used for western blotting.

### 2.3. Western Blotting

Cells that were treated with IGF-1, IL-6 and diosmetin were washed twice with ice-cold PBS and incubated on ice for 10 min in lysis buffer (50 mM NaCl, 50 mM pyrophosphate, 5 mM EDTA, 50 mM NaF, 5 mM EGTA, 0.1% Triton X-100, 100 μM Na_3_VO_4_, 10 mM HEPES pH 7.4, 10 μg leupeptin/ml and 1 mM phenylmethylsulfonyl fluoride). Total cell lysates were collected after scraping the cells, which were then centrifuged at 12,000× *g* for 10 min at 4 °C. Protein concentrations were estimated using a Bradford assay (Bio-Rad Laboratories, Inc., Hercules, CA, USA), following which 25 μg protein samples were separated by 10% SDS-PAGE and transferred onto nitrocellulose membranes. The membranes were then blocked with 5% non-fat dry milk in PBS containing 0.1% Tween 20 for 1 h, before they were incubated with primary antibodies overnight at 4 °C, followed by incubation with secondary antibodies for 1 h at room temperature. Based on antibody protocols we made dilutions and used them to probe the nitrocellulose membrane. Protein bands were visualized using chemiluminescence substrates (Amersham; Cytiva, Amersham, UK). Among the antibodies used, anti-phosphorylated (p)-AKT (Ser-473; cat. no. 9277), anti-p-AKT (Thr-308; cat. no. 2965), anti-Rictor (cat. no. 2114), anti-p-Rictor (Thr-1135; cat. no. 3806), anti-cleaved caspase-3 (cat. no. 9661) and anti-p-ERK1/2 (Thr-202/Tyr-204; cat. no. 4377) antibodies were purchased from Cell Signaling Technology, Inc., Danvers, MA, USA. Anti-Bax (cat. no. sc-493), anti-p-PKCα (Ser-657; cat. no. sc-12356), anti-Bcl2 (cat. no. sc-7382) and anti-GAPDH (cat. no. sc-166545) antibodies were purchased from Santa Cruz Biotechnology, Inc., Dallas, TX, USA.

### 2.4. Statistical Analysis

Experiments were performed more than three times. Statistical differences among the treatment and control groups were determined using simple analysis of variance followed by multiple comparison tests (such as a post hoc Fisher’s test). A two-way ANOVA with multiple comparisons was also performed, while comparisons between two groups were determined using a paired Student’s *t*-test. All tests were two-tailed and *p* < 0.05 was considered to indicate a statistically significant difference.

## 3. Results

### 3.1. Dose- and Time-Dependent Response of Prostate Cancer Cells to IGF-1 Treatment and Subsequent Regulation of the RICTOR Pathway

**Figure 1:**
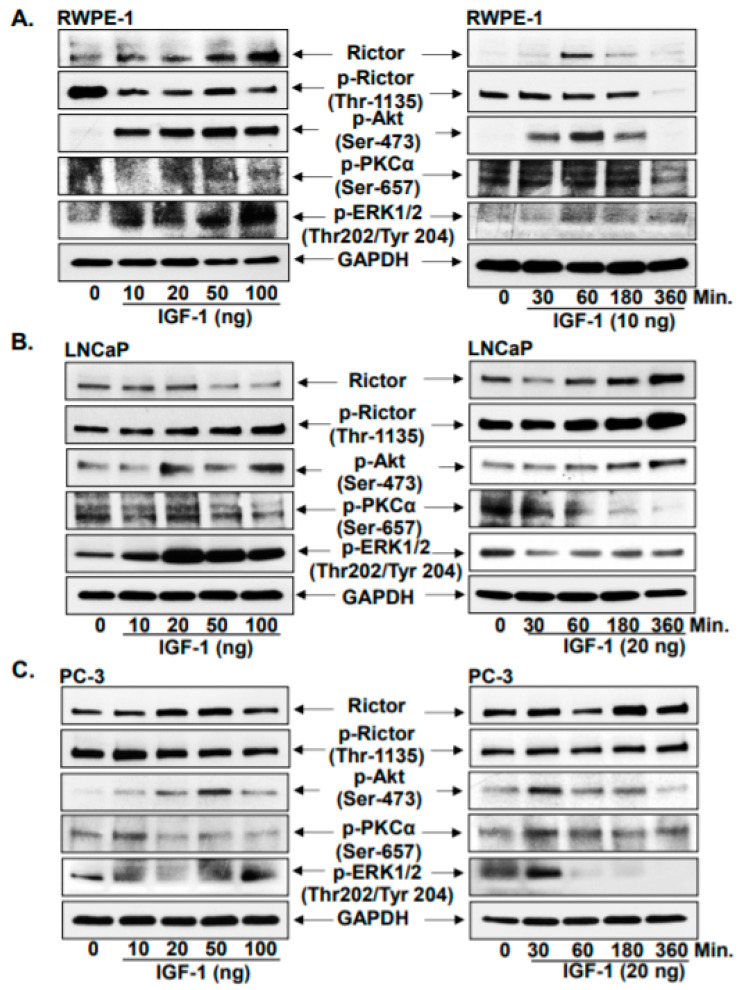
Growth factor (IGF-1) induced Rictor signaling cascade. Asynchronous (**A**) RWPE-1 normal prostate epithelial cells, (**B**) LNCaP (androgen sensitive), and (**C**) PC-3 (androgen refractory) cells were treated with IGF-1 in increasing concentrations (10, 20, 50, 100 ng) and time (30, 60, 180, 360 min) exposure, and later subjected to total cell lysate and western blot analysis. Protein resolved in SDS page gels were Rictor, p-Rictor (Thr-1135), p-AKT (Ser-476), p-PKCα (Ser-657), and p-ERK1/2 (Thr202/Tyr 204). Lanes mentioned as ‘0’ are PBS control-treated cells. For protein, control blots were stripped and reprobed with GAPDH.

Human normal prostate epithelial cells, RWPE-1, were first treated with increasing doses of IGF-1 (10, 20, 50 and 100 ng) for 8 h. It was observed that with the increasing dosage of IGF-1, Rictor expression was increased, whereas Rictor phosphorylation at Thr-1135 was reduced. AKT (Ser-473) and PKCα (Ser-657) phosphorylation was also found to be increased along with that of ERK1/2 phosphorylation (Thr202/Tyr 204). In RWPE1 cells, 10 ng IGF-1 was revealed to be the effective dose for increasing the expression of Rictor compared with that in the untreated control group. Therefore, 10 ng IGF-1 was subsequently used to treat RWPE1 cells for a range of different time points (30, 60, 180 and 360 min). Rictor expression was increased by as early as 30 min compared with that in the untreated control, but this increment was not significant. However, at 60 min after treatment, Rictor expression was found to be significantly increased, which was observed alongside reduced Rictor phosphorylation at Thr-1135 compared with that in the untreated control cells. In addition, treatment for 60 min was also sufficient to increase the phosphorylation of AKT (Ser-473), PKCα (Ser-657) and ERK1/2 (Thr202/Tyr204; Figure 1A). Based on these findings, the 60 min time point appears to be important for activating the Rictor signaling cascade.

Prostate cancer cell lines LNCaP (androgen responsive) and PC-3 (androgen refractory) were next treated with IGF-1 at a range of doses and for a range of time points. In LNCaP cells, it was observed that 20 ng IGF-1 was the dose that effectively increased Rictor expression and its phosphorylation at Thr-1135. In addition, IGF-1 (20 ng) was observed to significantly increase the phosphorylation of AKT (Ser-473) and PKCα (Ser-657). In terms of time dependency, a similar trend was observed in LNCaP cells as that observed in RWPE-1 cells, whereby the 60 min time point was effective in increasing the expression of Rictor, the levels of p-Rictor (Thr-1135), p-AKT (Ser-473) and p-PKCα (Ser-657), compared with those in untreated control cells (Figure 1B). Although Rictor was also activated in PC-3 cells following treatment with 20 ng IGF-1, this activation response occurred after 30 min, compared with 60 min in RWPE-1 and LNCaP cells (Figure 1C). This suggests that androgen refractory cells (PC-3) responded to IGF-1 earlier than RWPE-1 and LNCaP cells. This may be due to a higher metabolic rate in advanced-stage cancer cells compared with that in their early-stage counterparts.

### 3.2. Dose- and Time-Dependent Response of Prostate Cancer Cells to IL-6 Treatment and Subsequent Regulation of the Rictor Pathway

The next objective was to determine the potential role of IL-6, a proinflammatory cytokine, on the Rictor signaling pathway in prostate epithelial and cancer cells.

**Figure 2:**
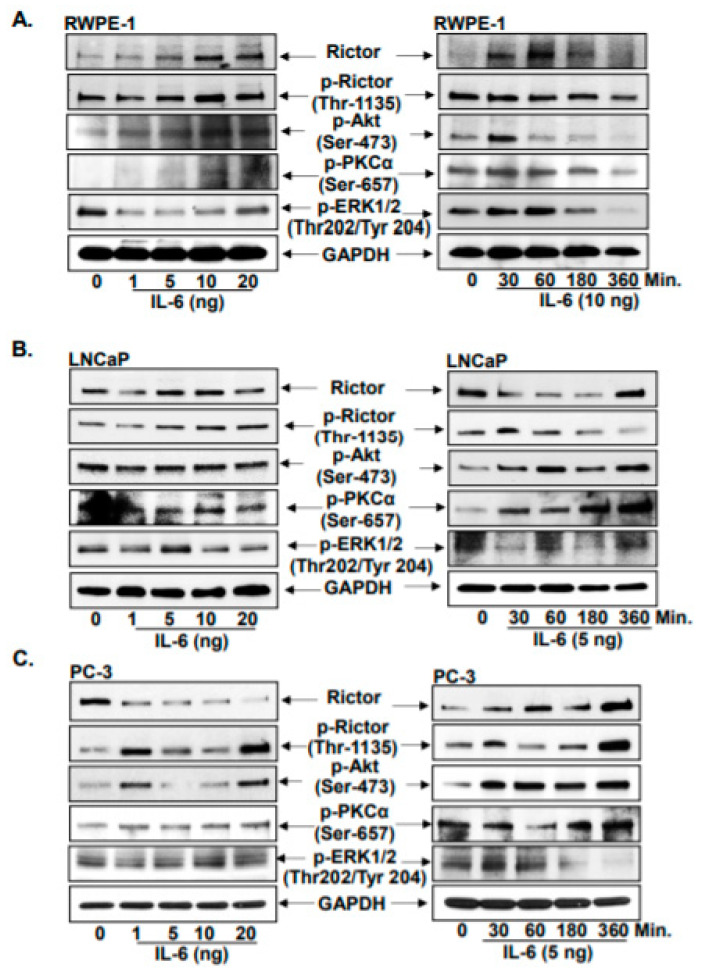
Pro-inflammatory cytokine IL-6 induced Rictor signaling cascade. Asynchronous (**A**) RWPE-1 normal prostate epithelial cells, (**B**) LNCaP (androgen sensitive), and (**C**) PC-3 (androgen refractory) cells were treated with IL-6 in increasing doses (1, 5, 10, 20 ng) and time (30, 60, 180, 360 min) exposure, and later subjected to total cell lysate and western blot analysis. Protein resolved in SDS page gels were Rictor, p-Rictor (Thr-1135), p-AKT (Ser-476), p-PKCα (Ser-657), and p-ERK1/2 (Thr202/Tyr 204). Lanes mentioned as ‘0’ are PBS control-treated cells. For protein, control blots were stripped and reprobed with GAPDH.

RWPE-1 cells were treated with increasing doses of IL-6 (1, 5, 10 and 20 ng) for 8 h. With increasing doses of IL-6, Rictor expression was increased. Similarly, Rictor phosphorylation (Thr-1135) also increased in the same manner. In addition, AKT (Ser-473) and PKCα (Ser-657) phosphorylation increased, while ERK1/2 phosphorylation (Thr-202/Tyr-204) decreased. IL-6 at 10 ng was the most effective dose modulating the expression of Rictor relative to untreated controls. Therefore, 10 ng of IL-6 was chosen to treat RWPE1 cells for subsequent time-course experiments (30, 60, 180 and 360 min). A significant increase in Rictor expression was observed at 30 min compared with that in the untreated control cells. Following 30 min of treatment with 10 ng IL-6, the Rictor phosphorylation (Thr-1135) also declined, and continued to decline in a time-dependent manner compared with that in the untreated control group. After treatment for 30 min, AKT phosphorylation (Ser-473) increased, as did PKCα (Ser-657) and ERK1/2 (Thr202/Tyr204) phosphorylation (Figure 2A). Data suggested that the 30 min time point could be important for activating Rictor signaling by IL-6.

Similarly, LNCaP cells and PC-3 cells were treated with varying doses of IL-6 (1, 5, 10 and 20 ng) at a range of time points (30, 60, 180 and 360 min). In terms of the IL-6 dose response, 5 ng was observed to be an effective dose, since Rictor and its phosphorylation (Thr-1135) were increased in both LNCaP and PC-3 cells. In terms of the time dependence, treatment of LNCaP cells with 5 ng IL-6 resulted in decreased Rictor expression and increased Rictor phosphorylation at Thr-1135 at 30 min. IL-6 at 5 ng was also observed to be the effective dose for increasing AKT (Ser-473) and PKCα (Ser-657) phosphorylation, which occurred within 30 min. By contrast, ERK1/2 (Thr202/Tyr204) phosphorylation was demonstrated to be decreased by treatment with 5 ng IL-6 (Figure 2B). In PC-3 cells, IL-6 was found to be effective at a lower dose of 1 ng after 8 h. Administration with 1 ng IL-6 significantly decreased the expression of Rictor, whilst significantly increasing the phosphorylation of Rictor (Thr-1135), AKT (Ser-473) and PKCα (Ser-657) compared with those in the untreated control group. No significant changes in p-ERK1/2 (Thr202/Tyr204) levels could be found after 1 ng IL-6 treatment (Figure 2C). PC-3 cells also responded to 1 ng IL-6 after 30 min, as indicated by the increased Rictor expression, in addition to the increased phosphorylation of Rictor (Thr-1135), AKT (Ser-473), PKCα (Ser-657) and ERK1/2 (Thr202/Tyr204; Figure 2). This suggests that androgen refractory (PC-3) cells are more sensitive to activation by IL-6. In addition, these observations suggest that prostate cancer cells are more sensitive to IL-6 than normal prostate epithelial cells. According to these findings, it could be implied that in patients with advanced-stage prostate cancer, altered levels of IL-6 may have a significant role in the Rictor signaling cascade to promote cancer cell survival through the AKT and PKCα pathways.

### 3.3. Silencing Rictor Regulates AKT and PKCα Phosphorylation

A previous study reported that silencing Rictor expression can abolish the phosphorylation of AKT and PKC [24]. In the present study, it was observed that silencing Rictor expression in PC-3 cells decreased AKT phosphorylation at Ser-473, whilst conversely increasing the phosphorylation of AKT at Thr-308 but not significantly changing the phosphorylation of PKCα at Ser-657. In addition, silencing AKT in PC-3 cells partially increased the phosphorylation of PKCα (Ser-657) in a non-significant manner. When AKT was overexpressed in DU145 cells, which do not have endogenous p-AKT (Ser-473), significantly increased Rictor expression and Rictor (Thr-1135) phosphorylation were observed. This suggests that a loop exists in the Rictor/AKT/PKCα axis, whereby in the absence of AKT, Rictor may have a role in increasing the phosphorylation of PKCα to promote cell survival and proliferation.

**Figure 3:**
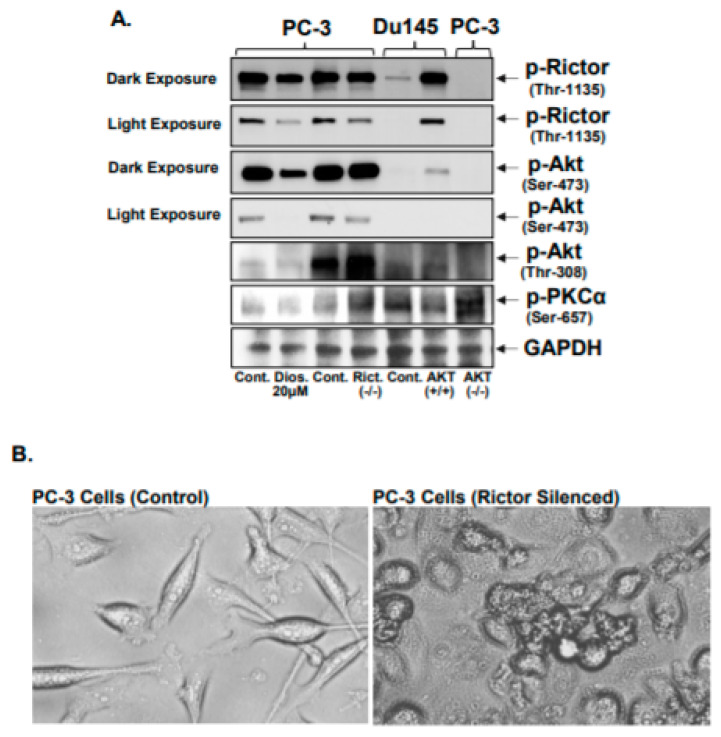
Effect of Rictor silencing in prostate cancer cells. Rictor (shRNA/ Lenti vector (Human) pGFP-iLenti) transfected in androgen-refractory PC-3 cells and Dominant Negative-AKT pUSEamp plasmid was transiently transfected to PC-3 cells, while DU145 cells were transfected with pLNCX vector containing AKT overexpression plasmid. After 48 h of transfection, total lysates were prepared. PC-3 cells were treated with 20 μM diosmetin for 24 h, all the treated total lysates were resolved in SDS Page gel to observe the altered p-AKT (Ser-476), p-PKCα (Ser-657), and p-Rictor (Thr-1135) (**A**). Lanes mentioned as ‘Cont’ are control (cell lysate). For protein, control blots were stripped and reprobed with GAPDH. Microphotographs of PC-3 cells with Rictor silenced cells (transient transfection) (**B**).

Diosmetin was found to be effective in inhibiting the phosphorylation of both AKT (at Ser-473 and Thr-308) and PKCα (Ser-657; Figure 3A) in PC-3 cells. This suggests that diosmetin has a broad spectrum effect in modulating AKT and PKCα activation in prostate cancer cells. In PC-3 cells where Rictor expression was knocked down, the shape of the cells changed, and became more spherical and smaller in size (Figure 3B). Previous reports also reported that conditional Rictor knockout mice exhibited altered cellular morphology and a reduction in cell size [25].

### 3.4. Dose Response Effects of Diosmetin Treatment on Rictor, AKT and PKCα

**Figure 4:**
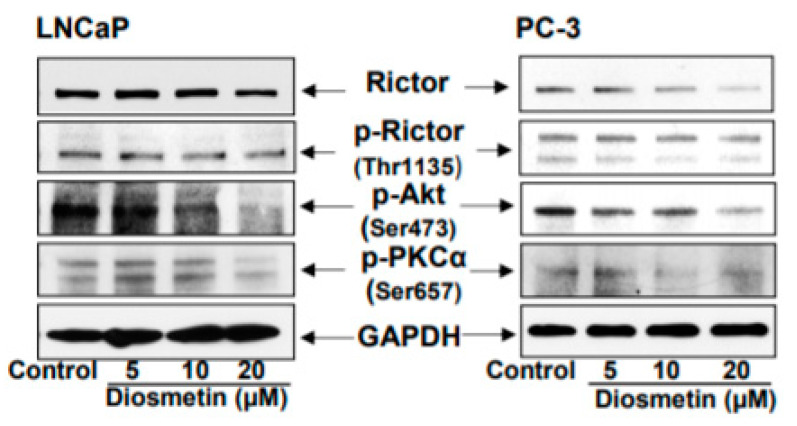
Diosmetin dose response treatment to prostate cancer cells inhibited Rictor, p-Rictor, p-AKT, and p-PKCα expression. LNCaP and PC-3 cells were treated with diosmetin in dose response (5, 10, and 20 μM) for 24 h, and total lysates were made to observe the altered expressions of Rictor, p-Rictor (Thr1135), p-AKT (Ser-476), and p-PKCα Ser-657. For protein, control blots were stripped and reprobed with GAPDH.

The next objective in the present study was to assess the effect of different doses of diosmetin on the Rictor signaling cascade in LNCaP and PC-3 cells. These two cell lines were treated with varying concentrations of diosmetin (5, 10, and 20 μM) for 24 h. The levels of Rictor expression, Rictor phosphorylation at Thr-1135, p-AKT (Ser-473) and p-PKCα (Ser-657) were all found to be decreased in a dose-dependent manner. No significant changes could be observed in Rictor expression in LNCaP cells, although at the higher dose of 20 μM, decreased Rictor expression was observed (Figure 4). These findings suggest that diosmetin has the potential to modulate the Rictor-induced phosphorylation of AKT and PKCα.

### 3.5. Potential Role of Diosmetin on Growth Factor- and Cytokine-Induced Rictor, AKT and PKCα Activation

**Figure 5:**
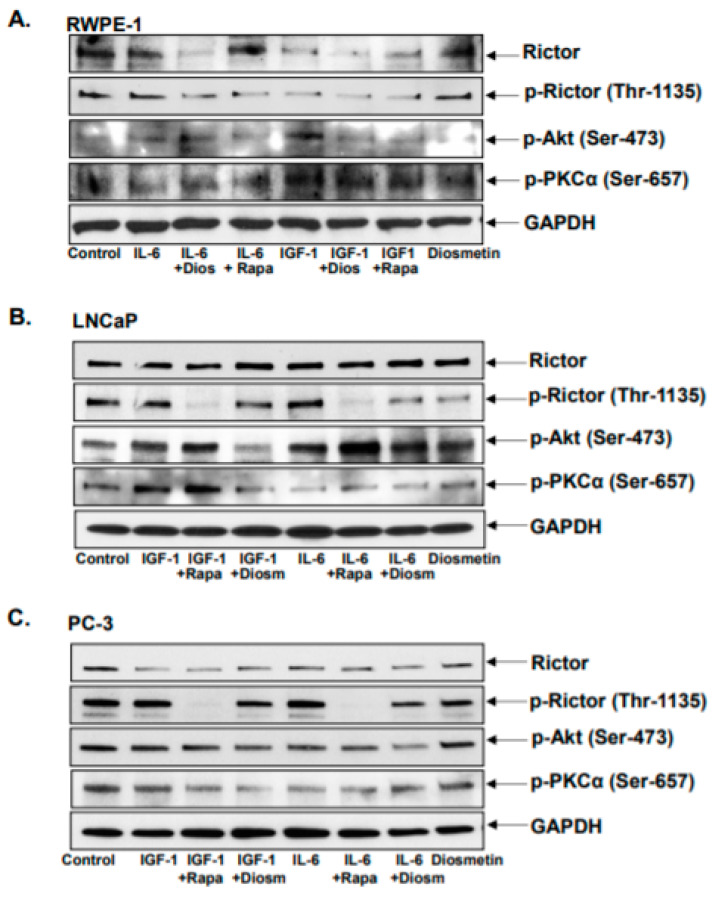
Combinatorial effect of diosmetin with IGF-1 and IL-6 in prostate cancer cells. LNCaP (**B**) and PC-3 (**C**) prostate cancer cells, and normal prostate epithelial cells (RWPE1) (**A**) were pretreated with Rapamycin (10 nM) and Diosmetin (20 μM) for 30 minutes. These cells were later treated with IGF-1 (20 ng concentration to LNCaP and PC-3, and 10 ng to RWPE1 cells) and IL-6 (5 ng concentration to LNCaP and PC-3 cells, and 10 ng to RWPE1 cells). After 24 h, total lysates were made to determine the protein expressions of Rictor, p-Rictor (Thr1135), p-AKT (Ser-476), and p-PKCα Ser-657. For protein loading, control blots were stripped and reprobed with GAPDH.

The growth factor IGF-1 and the cytokine IL-6 both exerted significant effects on Rictor as aforementioned. Therefore, the role of diosmetin on IGF-1- and IL-6-induced Rictor signaling activation was next assessed. RWPE1 cells were pre-treated with 10 ng IGF-1 and 10 ng IL-6, whilst LNCaP and PC-3 cells were treated with 20 ng IGF-1 and 5 ng IL-6 for 1 h. After the 1 h treatment, diosmetin treatment was administered (10 μM for RWPE1 cells; and 20 μM for LNCaP and PC-3 cells) for 24 h. The culture media that was used was charcoal-stripped and devoid of any growth factors. The effects mediated by IGF-1 and IL-6 pre-treatment were found to be attenuated by subsequent treatment with diosmetin. The expression of Rictor, phosphorylation of Rictor (Thr-1135), AKT (Ser-473) and PKCα (Ser-657) were all increased after IGF-1 and IL-6 treatment in both prostate cancer cell lines (Figure 5B,C) and in RWPE1 cells (Figure 5A). By contrast, post-treatment with diosmetin significantly reduced the levels of Rictor expression, Rictor (Thr-1135), AKT (Ser-473) and PKCα (Ser-657) phosphorylation in all cell lines tested. Rapamycin, which is an inhibitor of mTOR, was unable to suppress Rictor expression or reduce AKT (Ser-473) and PKCα (Ser-657) phosphorylation, although it could inhibit Rictor (Thr-1135) phosphorylation. Additionally, combining rapamycin with IGF-1 and IL-6 treatment almost completely inhibited Rictor (Thr-1135) phosphorylation. By contrast, the inhibitory effects of diosmetin on the inhibition of Rictor (Thr-1135) were found to be moderate. A previous report suggested that the phosphorylation state of Rictor within the mTORC2 complex, specifically at the Thr-1135 residue, could be considered to be an indirect activity marker of this kinase complex [26]. These findings suggest that diosmetin has the potential to reverse the increased expression of Rictor and increased phosphorylation of AKT (Ser-473) and PKCα (Ser-657) after IGF-1 and IL-6 treatment. In addition, diosmetin may serve to be an effective agent for targeting the Rictor cascade to inhibit prostate cancer progression.

### 3.6. Dose Response Effects of Diosmetin on the Apoptotic Machinery

**Figure 6:**
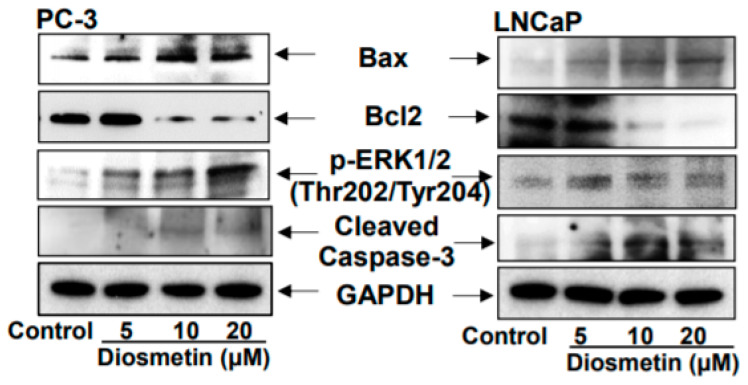
Diosmetin dose response effect on BCl2, Bax, Cleaved caspase-3 and ERK1/2 protein expression. Prostate cancer, LNCaP and PC-3, cells were treated with diosmetin (5, 10, 20 μM) for 24 h. Total lysates were made to observe the altered expressions of BCl2, Bax, Cleaved caspase-3 and ERK1/2. For protein, control blots were stripped and reprobed with GAPDH.

Next, the effect of various doses of diosmetin (5, 10 and 20 μM) on LNCaP and PC-3 cell apoptosis was assessed. In both LNCaP and PC-3 cells, increased Bax expression and ERK1/2 phosphorylation were observed, whilst Bcl2 expression was significantly decreased (Figure 6). Additionally, increased cleaved caspase-3 expression was observed following diosmetin treatment in a dose-dependent manner in PC-3 cells and LNCaP cells. This suggests that the apoptotic machinery was activated in prostate cancer cells after diosmetin treatment.

## 4. Discussion

The mTOR pathway is a major regulator of mammalian metabolism and physiology, and is commonly dysregulated in a variety of human malignancies. Recent reports suggest that the PI3K/AKT pathway is one of the major pathways that can regulate mTOR [27] and MAPK or ERK1/2 signaling [28]. In addition, a key role for the PI3K/AKT/mTOR signaling cascade in the development of castrate resistant prostate cancer (CRPC) has been previously proposed [29]. This pathway, which integrates growth signals to promote prostate cancer cell proliferation, differentiation, metabolism and survival, is known to be dysregulated in advanced stages of prostate cancer [29]. Previous pre-clinical studies have demonstrated that a connection exists between AKT/mTOR signaling and androgen receptor activation, suggesting a functional relationship between these two major pathways in CRPC [30].

PKC and AKT kinases can regulate cell proliferation, differentiation, survival and apoptosis [29]. Protein kinase C activity is controlled by phosphorylation in the activation loop (A-loop) within the kinase domain, specifically in the turn and hydrophobic motifs of the C-terminal region [24,31]. The cryo-electron microscopy structure of mTORC2 indicates that the mTORC2 complex is comprised of Rictor, mammalian lethal with SEC13 protein 8, Sin1 and mTOR [32]. Previous reports suggest that silencing Rictor expression can reduce the phosphorylation of AKT at the hydrophobic motif [33,34,35], whilst also reducing the phosphorylation of PKCα [33,36]. These observations suggest that Rictor can regulate PKC and AKT activity. Growth factor-induced activation of mTORC2 has been associated with Rictor phosphorylation at the Thr-1135 residue, such that oncogenic forms of Ras and PI3K can phosphorylate Rictor at Thr-1135 [26]. However, introduction of mutations that prevent Rictor phosphorylation in Rictor-deficient mouse embryonic fibroblasts could not alter AKT phosphorylation in the presence of growth factors, suggesting that Rictor phosphorylation at Thr-1135 is not an absolute necessity for mTORC2 kinase activity [26]. In addition, previous reports suggested that a Rictor phosphorylation mutant (Thr-1135) substitution in Rictor-null and wild-type cells increased mTORC2-dependent AKT phosphorylation (Ser-473), though the specific molecular mechanism remains unclear [37].

Diosmetin is a natural plant flavone that has been found to exhibit antioxidant, anti-inflammatory and antitumor effects [22]. It was previously demonstrated that diosmetin can induce S-phase and G_1_-phase cell cycle arrest in PC-3 and LNCaP cells, respectively [23]. Diosmetin treatment of PC-3 cells dose-dependently increased cell apoptosis by increasing the Bax/Bcl-2 ratio, upregulating the expression of apoptosis-related proteins and subsequently activating caspase-3. Bax is a pro-apoptotic protein that shuttles back into the mitochondria after the induction of apoptosis to depolarize the mitochondrial potential. By contrast, Bcl-2 is an anti-apoptotic protein that alters Bax functioning [38]. Therefore, the ratio of Bax/Bcl-2 is considered to be an important indicator of apoptosis [39]. Diosmetin has been previously reported to suppress tumor cell proliferation and migration, whilst inducing apoptosis in B16F10 melanoma cells [40]. In non-small cell lung cancer cells, diosmetin treatment can also induce apoptosis and enhance the chemotherapeutic efficacy of paclitaxel [41].

In a previous study, diosmetin was not shown to affect AKT, ERK1/2 or AMPK expression, but it did reduce the phosphorylation of AKT and ERK1/2 whilst inhibiting mTOR expression [42]. In the present study, it was observed that diosmetin treatment inhibited the phosphorylation of AKT at Ser-473 and Rictor at Thr-1135 in LNCaP and PC-3 cells after treatment with or without IGF-1 and IL-6. Therefore, diosmetin may modulate the activity of these key molecules to regulate the progression of prostate cancer.

It was also observed that both IGF-1 and IL-6 can induce the phosphorylation of AKT at Ser-473 and PKCα at Ser-657 in prostate cancer cells in the present study. Downstream of mTORC2, mTORC2 can regulate AKT activation through phosphorylation at Ser-473 [34,43,44]. Full activation of AKT is required to regulate cell proliferation, cell survival and migration [43]. Conditional Rictor knockout in the mouse central nervous system resulted in altered cellular morphology and reduction in cell size, coupled with the loss of PKC, AKT and SGK1 activation. However, no change in mTORC1 activity was observed [25]. In the present study, PC-3 cells were observed to be smaller and round in shape after Rictor silencing.

Rictor may be the major player in the regulation of PKCα and AKT. Previous reports suggest that Rictor silencing can reduce the phosphorylation of PKCα [45]. The present study found that Rictor silencing in prostate cancer PC-3 cells resulted in no significant change in phosphorylation of PKCα at Ser-657 and decreased phosphorylation of AKT at Ser-473. The phosphorylation of PKCα at Ser-657 was more pronounced when AKT expression was silenced in PC-3 cells. These findings suggest that substrate phosphorylation and PKC kinase activity were altered in Rictor knockdown PC-3 cells. Rictor knockdown in HeLa cells resulted in reduced PKC kinase activity, which was consistent with reduced phosphorylation at Ser-657 and PKCα kinase activity [36,46]. Previously, another report also found that Rictor silencing in various human cancer cell lines could reduce the phosphorylation of AKT at Ser-473, which increased the kinase activity of Thr-308 on AKT by 4-5-fold [47]. The present study observed increased Thr-308 expression when Rictor was silenced in PC-3 cells, which possibly revealed the function of Rictor in the regulation of AKT and PKC, which is promoting phosphorylation and kinase activity.

Phosphorylation of Ser-657 in the hydrophobic motif of all PKC isotypes requires mTORC2 [24,36]. PKCα phosphorylation at Ser-657 can be constitutive and autophosphorylated [48]. A previous report suggested that the AKT consensus sequence around Thr-308 (T^308^FCGT) remained fully conserved in the PKC-α activation loop [49]. This may explain why following Rictor silencing, PKCα phosphorylation at Ser-657 and AKT phosphorylation at Thr-308 were both increased to compensate for the reduction of AKT phosphorylation at Ser-473, which may facilitate cell survival.

The present study observed that in prostate cancer cells, silencing Rictor expression strongly inhibited AKT phosphorylation at Ser-473 but increased PKCα phosphorylation at Ser-657. Furthermore, diosmetin treatment resulted in the reduction of PKCα (Ser-657) and AKT (Ser-473) phosphorylation. Diosmetin treatment also reversed the IGF-1- and IL-6-induced phosphorylation of both PKCα and AKT. Therefore, this suggests that diosmetin has the potential to modulate both AKT and PKCα phosphorylation, which may inhibit prostate cancer cell proliferation and survival. Manipulating Rictor activity instead of knocking out Rictor expression appears to be a more efficacious strategy, since the loss of Rictor expression can lead to immunodeficiency, and impaired antibody production and autoimmune responses [50]. Diosmetin can manipulate the activity of Rictor by inhibiting AKT (Ser-473) and PKCα (Ser-657) phosphorylation, thereby inhibiting cell proliferation and survival.

**Figure 7:**
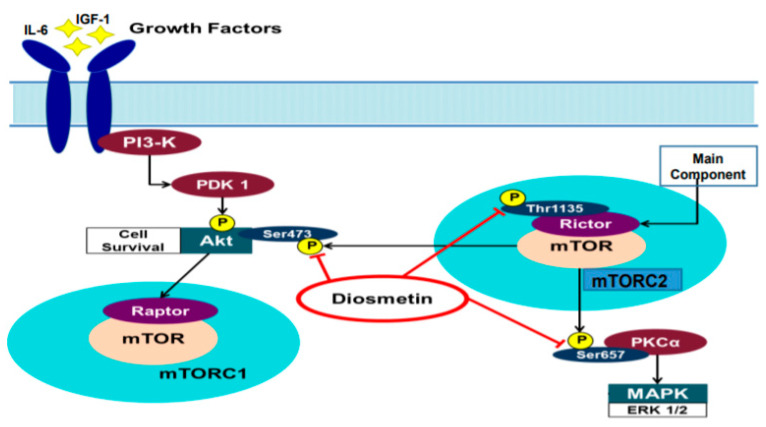
Schematic representation of Rictor-AKT–PKCα pathways and their interactions with each other in forming a growth promoting loop. Diosmetin was able to modulate these molecules by inhibiting their phosphorylation to inhibit the progression of prostate cancer.

The present study demonstrated that diosmetin is able to effectively alter the AKT and PKCα signaling cascade in prostate cancer cells, which can be activated by both IGF-1 and IL-6. Diosmetin treatment may modulate prostate cancer cell activation by IL-6 and IGF-1 and induces apoptosis downstream of Rictor signaling (Figure 7). To conclude, these findings suggested that diosmetin may have potential as a therapeutic option for prostate cancer.

## Data Availability

All data generated or analyzed during this study are included in this published article.
